# Supramolecular order controls the rotation frequency of artificial molecular motors

**DOI:** 10.1039/d6sc03692a

**Published:** 2026-07-29

**Authors:** Alexander Ryabchun, Manee Patanapongpibul, Federico Lancia, Dmitry Morozov, Jiawen Chen, Ben L. Feringa, Nathalie Katsonis

**Affiliations:** a Stratingh Institute for Chemistry, University of Groningen Nijenborgh 8 9747AG Groningen The Netherlands n.h.katsonis@rug.nl; b Department of Chemistry and Nanoscience Center, University of Jyväskylä P. O. Box 35 40014 Jyväskylä Finland

## Abstract

Artificial molecular motors convert light into rotary motion and are central components of synthetic molecular machineries. Their rotation frequency is key to their performance and is typically rationalized in terms of intrinsic molecular structure and solvent viscosity, while the influence of supramolecular organization remains largely unexplored. Here, we show that motor rotation is governed not only by viscosity but also by the supramolecular organisation of the surrounding medium. Thermodynamic analysis reveals that, beyond the viscosity-related increase observed in the isotropic phase of the same host, nematic order introduces an additional enthalpic penalty of 10–14 kJ mol^−1^ for the rate-determining thermal helix inversion. The magnitude of this penalty follows the alignment of the metastable *cis*-states involved in helix inversion, consistent with an order-dependent elastic resistance. These findings identify supramolecular order as a kinetic control parameter for artificial molecular motors and reveal reciprocal coupling between molecular-machine operation and soft-matter organization.

## Abstract 2

The long-range order of liquid crystals controls how fast light-driven molecular motors rotate, identifying soft-matter organization as a kinetic control parameter in the design of artificial molecular machines.

## Graphical abstract

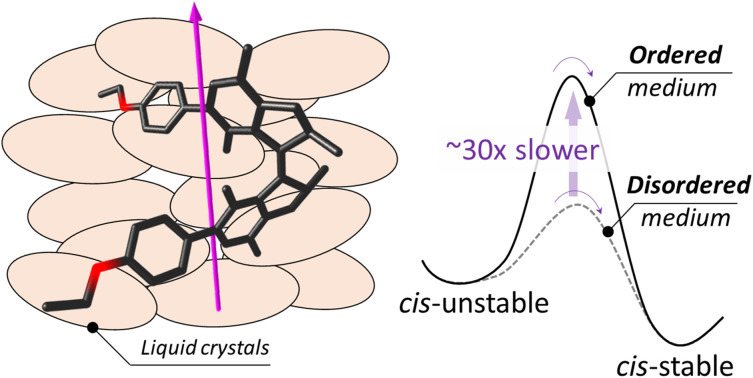

## Introduction

Artificial molecular motors convert light into rotary motion^1–8^ and represent one of the most advanced classes of synthetic molecular machines.^9–15^ Their controlled rotation under illumination has enabled functions ranging from catalysis^16^ and chiral amplification,^17,18^ to actuation in soft materials^19–24^ and optics.^25–28^ Two parameters are central to their operation: their coupling to surrounding matter, which determines how molecular motion is transmitted across length scales, and their rotation frequency, which sets the kinetics of the induced changes in the soft molecular matter in which they are embedded. While the effects of motor rotation have been extensively explored in organized media, the rotation frequency itself has largely been rationalized in terms of intrinsic molecular structure and solvent viscosity.^29^ Most studies of molecular motor rotation have focused on common organic solvents, where rotation slows with increasing viscosity.^16–20^ However, many practical implementations embed motors within supramolecular soft matter systems,^30–32^ raising a fundamental question: can the supramolecular organization of the surrounding medium directly influence the operating speed of an artificial molecular machine?

Artificial overcrowded alkene motors operate through a four-step rotary cycle comprising two photochemical isomerization and two thermal helix inversion (THI) steps. While the photoisomerization steps proceed rapidly upon illumination, the THI steps involve activation barriers that determine the overall rotation frequency. In first-generation motors, the initial THI from the *cis*-unstable to the *cis*-stable state is typically rate-determining and therefore defines the timescale of rotation.

Nematic liquid crystals offer an ideal model system to investigate how orientational order influences molecular motion. In their nematic phase, the molecules exhibit long-range directional alignment while maintaining fluidity, allowing comparison between isotropic and anisotropic states within the same chemical medium. This provides a means to isolate the effect of anisotropic order from viscosity within a single chemical environment. This unique combination makes liquid crystals particularly suitable for probing how ordered soft matter environments influence molecular motion.^33^ While molecular motors incorporated in liquid crystals have been exploited for chiral amplification,^17,18^ colour modulation,^26–28^ and macroscopic actuation,^19,21,24^ these studies have primarily focused on collective effects rather than on how nematic order modulates the intrinsic rotation frequency of the embedded motors. Thus, whether long-range orientational order can directly regulate the intrinsic kinetics of molecular motors remains unknown.

We have investigated a series of first-generation overcrowded alkene molecular motors bearing systematically varied substitution patterns, in order to address this question. By modifying the position and rigidity of lateral substituents, we tuned the molecular anisotropy of the motors and, consequently, their alignment within a nematic liquid crystal host. Polarized UV-vis spectroscopy was used to quantify motor orientation through dichroic order parameters, while temperature-dependent kinetic measurements enabled thermodynamic analysis of the rate-determining thermal helix inversion step in both isotropic and nematic phases of the same medium. This approach enables direct correlation between motor alignment and rotation frequency and allows testing whether supramolecular order acts as an independent kinetic control parameter for artificial molecular motors.

## Results and discussion

### Motor operation is preserved in a nematic host

**Fig. 1:**
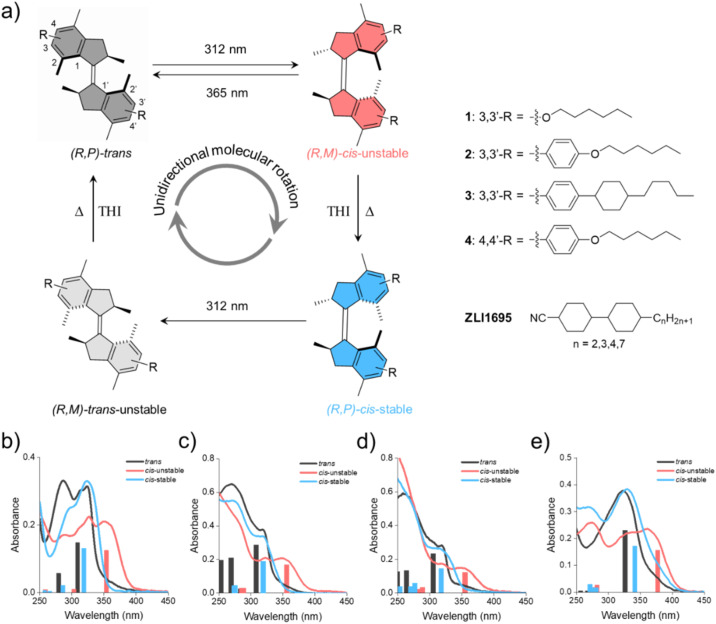
Rotary cycle and spectroscopic operation of molecular motors in a nematic host. (a) Four-step rotary cycle of light-driven molecular motors 1–4. Only the *R*-enantiomer is shown (left panel). Chemical structure of liquid crystal host ZLI1695 (right panel). (b–e) Absorbance spectra of different states of molecular motors 1–4 in liquid crystal, respectively. The spectra were obtained at room temperature, corresponding to the nematic phase of the liquid crystal host. The *cis*-unstable state of the motors was prepared by exposing the *trans*-stable form to 312 nm UV light. Bars in figures (b–e) correspond to the most intense electronic transitions as calculated by TD-DFT.

Four first-generation overcrowded alkene motors bearing distinct substitution patterns were synthesized to modulate molecular anisotropy and host–guest interactions (Fig. 1a). The motors were dispersed in the nematic liquid crystal ZLI1695, chosen for its well-defined nematic-to-isotropic phase transition that occurs at *T* ≈ 72 °C, which means that it becomes possible to compare the frequency of motor rotation in ordered and disordered states of exactly the same chemical medium. UV-vis spectroscopy confirms that all motors undergo the characteristic photochemical isomerization cycle within the liquid crystalline host (Fig. 1b–e), indicating that the crowded and anisotropic environment does not inhibit their operation.

The absorption spectra of motors 1–4 exhibit characteristic state-dependent shifts, most notably a significant bathochromic shift in the *cis*-unstable states (>355 nm). All states of motor 4 demonstrate a red shifted absorption due to its electron-donating alkyloxy-substituents. These experimental trends are corroborated by TD-DFT calculations, which identify the primary red-shifted peaks as π → π* (HOMO–LUMO) transitions.

Having established that motor operation is preserved in the liquid crystalline host, we next examine how molecular alignment is influenced by the substitution of the motor molecules.

### Substitution pattern determines motor alignment in nematic order

As mentioned above enantiomerically pure molecular motors are one of the most effective chiral molecules to induce cholesteric mesophase with widely tuneable optical properties.^25,27,28,34^ As we have previously pointed out,^25^ the orientation of the molecular motors in the liquid crystal host determines the optical properties of the cholesteric helices, and their propensity to amplify molecular motion across length scales. However, to date there are no studies shedding light on the orientation of molecular motors in organized liquid crystals environment in contrast to extensively studied photoswitches and dyes.^35–37^ We have thus investigated the alignment of the motor by using racemic mixtures of motors, in order to prevent the formation of a cholesteric phase. The dichroic order parameter (*S*_φ_) was calculated from polarized absorbance measurements (eqn (1)) to quantify motor alignment relative to the nematic director.^36,37^

1 *S*_φ_ = (*A*_‖_ − *A*_⊥_)/(*A*_‖_ + 2*A*_⊥_) = *S*_θ_*S*_β_

where *A*_‖_ and *A*_⊥_ – the absorbance of light polarized parallel and perpendicular to the liquid crystal alignment direction (*n*). Details of the formalism and decomposition of *S*_φ_ are provided in the SI.

**Fig. 2:**
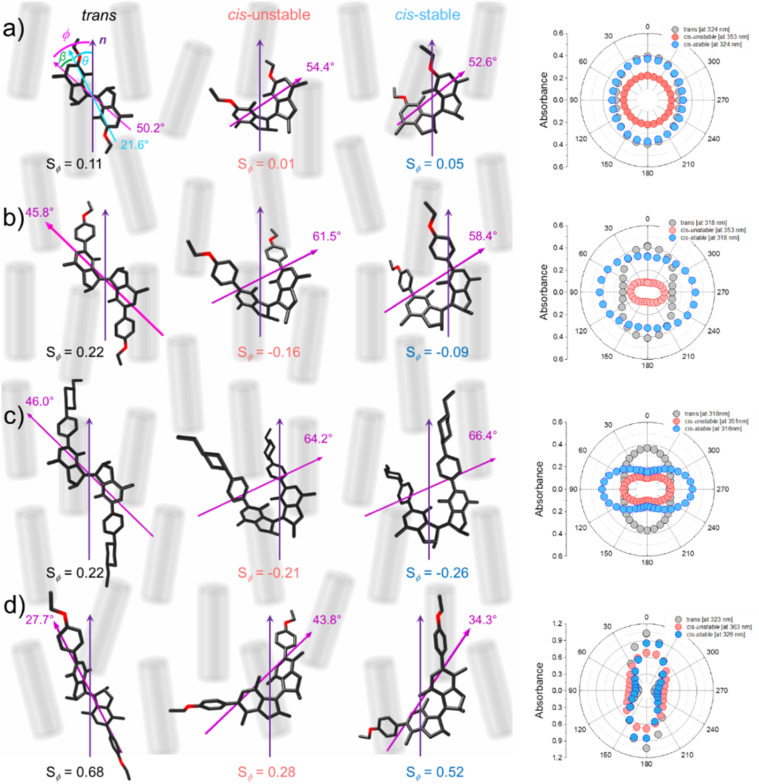
Structure-dependent alignment of molecular motors in nematic order. (a–d) Alignment of motors 1–4 as racemic mixtures, embedded into a nematic liquid crystal environment. In the optimized structures of motors 1–4, the calculated TDM (purple arrow), MOI (light blue) are shown. Details for the calculations are shown in the SI. The molecular motors are represented in respect to the molecular alignment of liquid crystal molecules (gray cylinders in the background, and violet arrow, *n*), according to the experimental data obtained by linearly polarized UV spectroscopy, that is plotted on the polar plots (right panels). Dichroic order parameter values (*S*_φ_) are provided in the figure and reflect the degree of alignment of the motor molecules, where 0 is disorder, 1 is perfect parallel alignment, and −0.5 is perfect alignment perpendicular to *n*. The aliphatic tails of motors have been shortened in sake of representation. (*R*)-Enantiomers of the motors are shown.

Fig. 2 summarizes the alignment behaviour of the molecular motors 1–4. The angular dependence of the polarized absorbance maximum allows estimating the location of the transition dipole moment (TDM) within the molecule and, consequently, the orientation of each molecular motor with respect to the liquid crystal molecules. The calculated TDMs and principal molecular axes are shown alongside the experimental polar plots.

Interestingly, motor 1 exhibits alignment of its *trans*-stable state along the nematic director (Fig. 2a). At the same time, the *cis*-stable and *cis*-unstable states are randomly aligned with the nematic director, which we associate with the low anisometry of the molecular shape. In contrast, motors 2 and 3 display pronounced and state-dependent alignment: their *trans*-states align parallel to the director, while their *cis*-states adopt preferential orientations that facilitate interaction of the rigid biphenyl substituents with the host matrix. Motor 4 in the *trans*-state displays the highest degree of orientation within the series (*S*_φ_ ≈ *S*_θ_ ∼ 0.68) along the liquid crystals, which corresponds to the best examples of dichroic dyes in liquid crystalline matrices.^38^ The high value of the order parameter is related to the long rod-like and rigid shape of the molecule. Both *cis*-states of motor 4 also have a predominant orientation as shown in Fig. 2d, but with significantly lower order parameter likely due to their bent-shape.

Across the series, the degree of alignment increases systematically from motor 1 to motor 4. We attribute this alignment dependence to the increased molecular anisotropy induced by the substitution pattern, as increased molecular anisotropy facilitates alignment of the motors in the nematic liquid crystal. These results establish a tunable structure–alignment relationship across the series and provide a direct spectroscopic handle on motor–host coupling before examining its kinetic consequences.

### Nematic order increases the activation barrier of thermal helix inversion

The thermal helix inversion (THI) is the rate-determining step in the rotation cycle of first-generation motors (Fig. 1a). Because this step involves a substantial conformational reorganization of the motor, its activation barrier defines the overall rotation frequency. To assess the influence of the orientational order on the rotation, we determined the activation parameters for the first thermal helix inversion (*cis*-unstable to *cis*-stable) in heptane, in the isotropic phase of the liquid crystal host, and in its nematic phase.

**Fig. 3:**
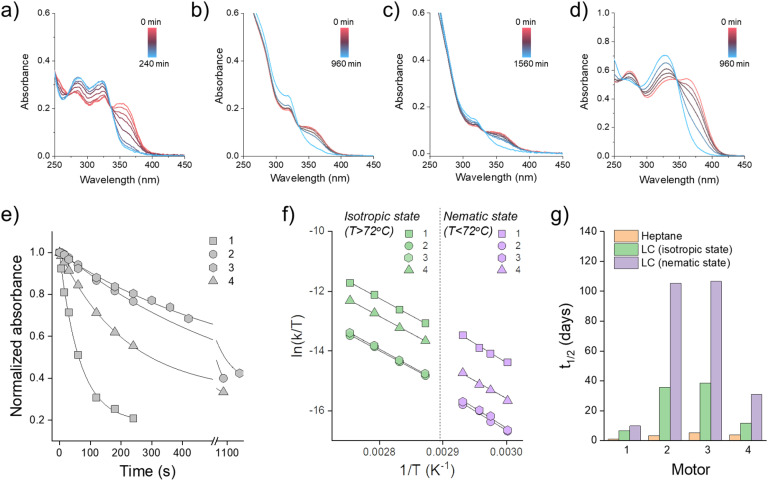
The nematic order enhances the activation barrier of thermal helix inversion. (a–d) Typical absorbance spectra of thermal helix inversion process (transition from *cis*-unstable- to *cis*-stable state) monitored in liquid crystalline nematic state at *T* = 63 °C. (e) Corresponding kinetic curves. (f) Eyring plots of motors 1–4 in liquid crystalline (*T* < 72 °C) and isotropic states (*T* > 72 °C). Vertical dashed line corresponds to the isotropization temperature of liquid crystal host. (g) Estimated half-life times (extrapolated to room temperature) of molecular motors 1–4 in heptane, in both the nematic and isotropic states of liquid crystal host.

These thermodynamic parameters were obtained from Eyring analysis^39^ (Fig. 3 and Table 1). The rates of thermal relaxation from *cis*-unstable to *cis*-stable were followed by UV-vis spectroscopy at the corresponding absorbance maxima (Fig. S2–S6). Fig. 3a–d show the absorbance spectra of motors 1–4, respectively, measured in the nematic state at 63 °C. One can see the drop of absorbance band at 350–400 nm which is associated with *cis*-unstable to *cis*-stable isomerization of motors. The values of Gibbs free energy activation (Δ^‡^*G*°), enthalpy (Δ^‡^*H*°), entropy (Δ^‡^*S*°) and half-life times of the *cis*-unstable states estimated using Eyring analysis are summarized in Table 1.

**Table 1:** Measured and calculated thermodynamic parameters of thermal helix inversion process (*cis*-unstable → *cis*-stable) of molecular motors 1–4 in different media. (N) and (iso) correspond to nematic and isotropic states of LC medium. The half-life time at 293 K were obtained by extrapolation of the Eyring fits. Values for LC (iso) are formal extrapolations used for comparison, since the host is nematic at 293 K

Motor	Medium	Δ^‡^*G*° (kJ mol^−1^)	Δ^‡^*H*° (kJ mol^−1^)	Δ^‡^*S*° (J K^−1^ mol^−1^)	*t*_1/2_ (days)
1	LC (N)	105.9 ± 0.3	106.9	3.5	9.8
	LC (iso)	104.9 ± 0.1	95.8	–31.1	6.6
	Heptane	100.0 ± 0.05	87.5	–42.9	0.9
	Calc.	97.5	91.0	–22.2	0.3
2	LC (N)	111.7 ± 0.4	109.2	–8.5	105.3
	LC (iso)	109.0 ± 0.2	94.9	–48.2	35.6
	Heptane	103.2 ± 0.06	90.4	–43.8	3.3
	Calc.	100.1	95.0	–17.7	0.9
3	LC (N)	111.7 ± 0.3	111.1	–2.1	106.6
	LC (iso)	109.2 ± 0.1	96.7	–42.6	38.6
	Heptane	104.4 ± 0.13	97.9	–22.2	5.3
	Calc.	104.2	93.0	–38.4	4.9
4	LC (N)	108.7 ± 0.2	105.2	–11.89	30.8
	LC (iso)	106.3 ± 0.1	95.2	–37.6	11.6
	Heptane	103.6 ± 0.09	96.9	–23.0	3.9
	Calc.	104.4	97.7	–22.8	5.3

The data show that all experimentally measured barriers in heptane go in line with ones predicted by DFT within a reasonable error margin of approx. 3 kJ mol^−1^ (Fig. S2–S6, and full energy diagram in Fig. S7). In heptane, the activation barriers follow the expected substitution trend, with motor 1 rotating fastest and motor 3 slowest, consistent with steric effects and DFT calculations. A similar influence of steric effects on rotation frequency was observed previously.^40^

Comparative analysis of energy barriers of THI in ordered nematic and disordered isotropic states of liquid crystal host (Fig. 3e and f and Table 1) revealed that all motors rotate slower in liquid crystal compared to heptane solution. When liquid crystals are in their isotropic state (no order), Δ^‡^*H*° is 1–8 kJ mol^−1^ higher compared to heptane which is likely associated with the fact that a nematic liquid crystal is more viscous than heptane [heptane – 0.6 mm^2^ s^−1^, ZLI1695 – 62 mm^2^ s^−1^].^41^ The effect of viscosity of solvent on rotation of the second generation motors was discussed in detail by Kistemaker *et al.*^29^

Strikingly, an additional increase in activation enthalpy of 10–14 kJ mol^−1^ is observed when the host transitions from the isotropic to the nematic phase (Fig. 3f). Because the chemical composition of the medium remains unchanged, this barrier enhancement cannot be attributed to viscosity alone and we conclude that it arises from the emergence of long-range orientational order.

The less negative values of Δ^‡^*S*° in the liquid crystal host indicate reduced reorganization of the nematic host, upon formation of the transition state (Fig. S8). Together with the increased Δ^‡^*H*°, this results in higher Δ^‡^*G*° values and consequently slower motor rotation. The magnitude of the additional barrier increase varies systematically across the motor series, demonstrating a direct correlation between motor alignment in the nematic field and the energetic cost of rotation.

Overall, the comparison across heptane, isotropic liquid crystal, and nematic liquid crystal resolves two distinct contributions to the rotational barrier: a viscosity-related increase upon moving from heptane to the isotropic host, and a second, larger enthalpic penalty that emerges only in the ordered nematic phase. Because the host chemistry is unchanged across the phase transition, this second contribution identifies orientational order as an independent determinant of motor kinetics. Together, these data indicate that the ordered supramolecular host reshapes the activation landscape.

### Alignment–barrier correlation reveals elastic coupling

The additional barrier enhancement in the nematic phase correlates with the degree of motor alignment determined from polarized absorption measurements.

Motors 2 and 3, whose *cis* states exhibit pronounced alignment relative to the nematic director, show the largest increase in activation enthalpy upon transition from the isotropic to the nematic phase. In contrast, motor 1, which shows weaker alignment of its *cis*-states, exhibits a smaller barrier increase. Motor 4 presents an intermediate behaviour, consistent with its alignment characteristics. These trends indicate that the energetic cost of thermal helix inversion depends on the extent of motor–host coupling established in the ordered medium.

These results indicate: (i) introduction of rigid substituents into the motor core slows down molecular rotation; (ii) the further elongation of lateral substituents does not significantly influence the rotation speed; and (iii) substitution position matters, so that, 3,3′-substituted motors rotate slower that 4,4-substituted motors. The effect of substitution is the most prominent, which probably correlates with the alignment motors in ordered liquid crystal environment.

As a computational study recently pointed out,^42^ the orientation of solvent molecules in the solvation shell of the motor impacts the energy landscape of its rotation cycle. Here, the alignment of liquid crystal molecules (the long molecular axis of liquid crystals) is parallel to the long and rigid substituents of the core of *cis*-states of motors 2 and 3 which provides the largest interactions of motors with their local environment (Fig. 2b and c). Therefore, the motor needs to overcome a higher energy barrier to oppose not only viscous forces^43^ but also to oppose the elasticity of the liquid crystalline phase (the forces preventing a local deformation of director field of liquid crystals).^44^ As a result, we conclude that the molecular order of liquid crystals additionally slows the rotation of the motors (Fig. 3g).

Overall, these trends show that substitution controls motor speed in the nematic host primarily by modulating motor–host coupling, not only by adding steric bulk. Motors 2 and 3, whose *cis* states align most effectively with the director, pay the largest enthalpic penalty for helix inversion. We attribute this to the need to disturb the locally ordered host during rotation, so that the motor must overcome not only viscous drag but also an order-dependent elastic resistance. Notably, this behaviour contrasts with azobenzene switches, whose thermal isomerization is accelerated in nematic hosts.^45^

### Slowed rotation in ordered media reveals a transient intermediate in the rotation cycle

**Fig. 4:**
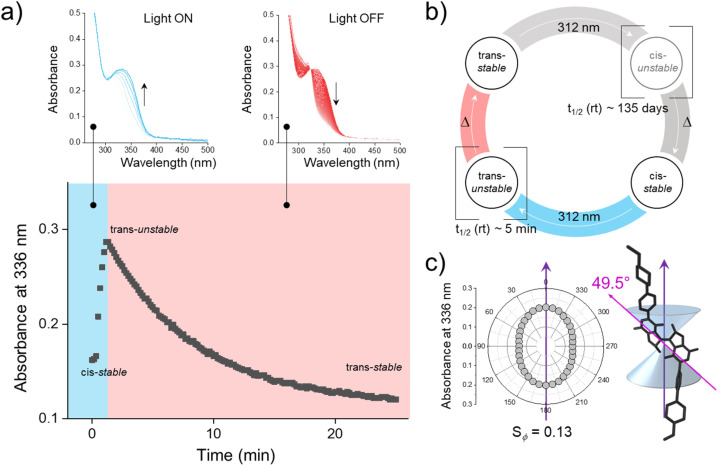
Nematic-induced slowing reveals the *trans*-unstable intermediate. (a) Absorbance spectra (top) of photo-induced *cis*-stable- to *trans*-unstable state transition followed by its thermal relaxation to *trans*-stable state of motor 3 and corresponding kinetic profiles (bottom) measured at room temperature. (b) Scheme of molecular rotation of motor 3 with indicated half-life times of both unstable states. (c) Polar plot of *trans*-unstable state of motor 3 embedded in unidirectional aligned liquid crystal environment indicating that motor is aligned along liquid crystal molecules (purple vertical arrow). *S*_φ_ – value of dichroic order parameter.

A direct consequence of the substantial slowing of motor rotation, is that it enables the observation of normally transient intermediates. Indeed, the barrier enhancement in the nematic phase slows motor rotation sufficiently to permit direct observation of the *trans*-unstable intermediate at room temperature (Fig. 4a) which is otherwise possible only at low temperatures.^24,46^ Upon photoexcitation of the *cis*-stable state of motor 3, once it is embedded in the nematic host, a distinct absorption band corresponding to the *trans*-unstable species emerges and decays thermally to the *trans*-stable state over approximately 20 min. Analysis of the kinetic trace yields a half-life of about 5 min at room temperature, corresponding to an activation barrier of 88.1 kJ mol^−1^ for the second thermal helix inversion. A comparable behavior is observed for motor 2 (Fig. S10), whereas the intermediate remains too short-lived to resolve for motors 1 and 4 under the same conditions.

Polarized absorption measurements indicate that the *trans*-unstable state of motor 3 remains preferentially aligned along the nematic director, albeit with a reduced order parameter relative to the *trans*-stable form (*S*_φ_ ≈ 0.13; Fig. 4c).

We conclude that the *trans*-unstable state becomes directly observable because the ordered host selectively slows its decay, consistent with appreciable coupling of this intermediate to the nematic environment. Supramolecular order therefore does more than tune a rate constant: it can qualitatively reshape the experimentally accessible rotary cycle by stabilizing intermediates that remain hidden in isotropic media.

## Conclusions

We show that the operating speed of artificial molecular motors is not solely encoded in molecular structure and solvent viscosity; it is also set by the orientational order of the surrounding medium. By comparing isotropic and nematic states of the same host, we isolate an order-dependent enthalpic penalty of 10–14 kJ mol^−1^ for the rate-limiting thermal helix inversion. Supramolecular order is therefore not just a passive environment for molecular motors, but an active kinetic control parameter. This principle should be relevant beyond thermotropic liquid crystals, in other anisotropic soft-matter environments where molecular machines operate under collective order.

## Author contributions

A. R. and N. K.: conceptualization. A. R.: investigation, methodology, formal analysis, visualization, writing the original draft. M. P. and D. M.: investigation, formal analysis. F. L. and J. C.: investigation, data curation. B. L. F. and N. K.: supervision and funding acquisition. N. K: project administration, review and editing.

## Conflicts of interest

There are no conflicts to declare.

## Supplementary Material

SC-OLF-D6SC03692A-s001

## Data Availability

The data supporting this article are included in the manuscript and supplementary information (SI). Supplementary information: optimized structures and calculated conformations are provided as a separate archive. See DOI: https://doi.org/10.1039/d6sc03692a.
