# Supplementary material for: Supramolecular order controls the rotation frequency of artificial molecular motors
Source: Chem Sci. 2026 Jul 29. Online ahead of print. doi: 10.1039/d6sc03692a (PMC13417791; doi:10.1039/d6sc03692a)
Supplement: SC-OLF-D6SC03692A-s001 [file SC-OLF-D6SC03692A-s001.pdf]

Supplementary Information (SI) for Chemical Science.
This journal is © The Royal Society of Chemistry 2026

Supporting Information

Supramolecular Order Controls the Rotation Frequency of Artificial Molecular Motors

Alexander Ryabchun[1], Manee Patanapongpibul[1], Federico Lancia[1], Dmitry Morozov[2], Jiawen Chen[1,3], Ben L. Feringa[1], Nathalie Katsonis[1]*

[1] Stratingh Institute for Chemistry, University of Groningen, Nijenborgh 8, 9747AG Groningen, The Netherlands

[2] Department of Chemistry and Nanoscience Center, University of Jyväskylä, PO Box 35, 40014 Jyväskylä, Finland

[3] Current address: Institute of Electronic Paper Displays, South China Academy of Advanced Optoelectronics, South China Normal University, Guangzhou, 510006, China

Corresponding e-mail: n.h.katsonis@rug.nl

Experimental part

Synthesis and characterization

General Remarks

Chemicals were purchased from Acros, Aldrich, Fluka or Merck and were used as received. Solvents for extraction and chromatography were technical grade. All solvents used in reactions were freshly distilled from appropriate drying agents before use. All reactions were performed under inert atmosphere (Ar). Analytical TLC was performed with Merck silica gel 60 F254 plates and visualization was accomplished by UV light. Flash chromatography was carried out using Merck silica gel 60 (230-400 mesh ASTM). NMR spectra were recorded on Agilent Technologies 400-MR (400/54 Premium Shielded) spectrometer (400 MHz). The deuterated solvents (CD_2Cl_2 and $CDCl_3$) were treated with Na_2CO_3, molecular sieves (4 Å) and degassed by argon prior to use. Chemical shifts are denoted in parts per million (ppm) relative to the residual solvent peak (CD_2Cl_2: ^{1}H δ = 5.32 ppm, ^{13}C δ = 53.84 ppm; $CDCl_3$: ^{1}H δ = 7.26 ppm, ^{13}C δ = 77.0 ppm). The splitting parameters are designated as follows: s = singlet, d = doublet, t = triplet, q = quartet, m = multiplet, dd = doublet of doublets. High-resolution mass spectrometry (ESIMS) was performed on a LTQ Orbitrap XL spectrometer with ESI ionization. The trans- and cis-forms of molecular motors 1, 2, 4 and functionalized motor core 5 were synthesized and purified as reported previously.[1,2] All motors were used as racemic mixtures. Compound 6 was synthesized and purified as reported previously.[3]

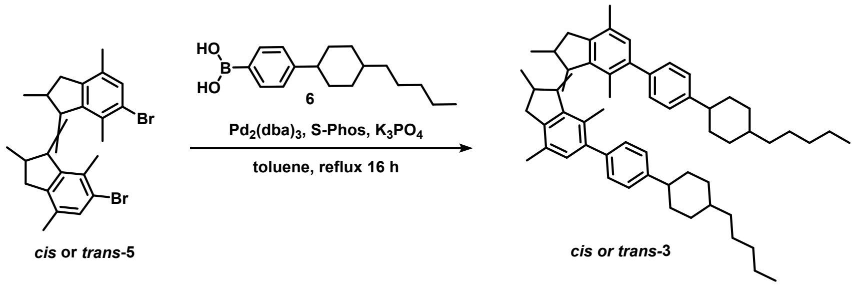

Scheme S1. Synthetic route to molecular motor **3**.

Motor 3

trans-**5** (474 mg, 1.0 mmol), $Pd_2(dba)_3$ (37.5 mg, 0.04 mmol), S-Phos (38 mg, 0.10 mmol), K_3PO_4 (636 mg, 3 mmol) and (4-(4-pentylcyclohexyl)phenyl)boronic acid (562 mg, 2.05 mmol) in toluene (15 mL) was heated at reflux for 16 h. After filtration, the solvent was evaporated and the residue was purified by flash column (SiO_2, heptane:dichloromethane = 9:1) to yield *trans*-**3** (473 mg, 50%) as a white solid. *Cis*-**3** was obtained following the same procedure.

Trans-**3**: ^{1}H NMR (400 MHz, CDCl3) δ 7.33 (d, J = 7.9 Hz, 2H), 7.26 (t, J = 4.0 Hz, 2H), 6.97 (s, 1H), 3.02 (q, J = 6.2 Hz, 1H), 2.77 (dd, J = 14.4, 5.6 Hz, 1H), 2.59 – 2.46 (m, 1H), 2.29 (d, J = 14.3 Hz, 4H), 2.23 (s, 3H), 2.02 – 1.85 (m, 4H), 1.52 – 1.43 (m, 1H), 1.30 (ddt, J = 26.2, 14.0, 7.1 Hz, 10H), 1.12 (d, J = 6.4 Hz, 3H), 1.05 (dd, J = 25.7, 14.1 Hz, 2H), 0.91 (t, J = 6.8 Hz, 3H). ^{13}C NMR (101 MHz, CDCl3) δ 146.24, 142.23, 142.19, 141.62, 141.27, 140.25, 131.14, 129.86, 129.56, 129.04, 126.59, 44.49, 42.58, 39.13, 37.59, 37.54, 34.60, 34.55, 33.84, 32.40, 26.84, 22.89, 21.49, 19.29, 18.37, 14.28.

HRMS (ESI-TOF) *m/z*: Calcd for $C_{58}H_{76}$ $[M + H]^+$ 772.59415; Found 772.59531.

Cis-**3**: ^{1}H NMR (400 MHz, $CDCl_3$) δ 7.29 – 7.16 (m, 4H), 6.92 (s, 1H), 3.44 – 3.38 (m, 1H), 3.13 (dd, J = 15.1, 6.3 Hz, 1H), 2.57 – 2.45 (m, 2H), 2.26 (s, 3H), 2.00 – 1.85 (m, 4H), 1.48 (s, 3H), 1.28 (ddd, J = 24.5, 13.2, 6.7 Hz, 10H), 1.18 – 1.12 (m, 3H), 1.10 – 1.02 (m, 2H), 0.90 (dt, J = 7.3, 3.6 Hz, 3H). ^{13}C NMR (101 MHz, $CDCl_3$) δ 146.13, 143.26, 142.04, 141.48, 140.37, 140.30, 130.88, 130.31, 129.76, 129.40, 126.47, 77.48, 77.16, 76.84, 44.48, 41.92, 38.87, 37.61, 37.56, 34.63, 34.55, 33.85, 32.41, 26.85, 22.89, 20.71, 19.79, 18.45, 14.28.

HRMS (ESI-TOF) *m/z*: Calcd for $C_{58}H_{76}$ $[M + H]^+$ 772.59415; Found 772.59504.

Sample preparation

The commercial mixture of bicyclohexane derivatives ZLI1695 (Merck) was used as the nematic liquid crystals host. The mixture becomes isotropic at 70–71°C. The motor-doped liquid crystal mixtures were prepared by dissolving of the motors (1 wt.%) and ZLI1695 in dichloromethane, followed by solvent evaporation and drying in vacuum. Next, the mixtures were introduced into sandwich-like quartz cells by capillary forces at room temperature. The home-made quartz cells of 10 μm thickness were used. Inner surfaces of quartz substrates were coated with polyimide (SUNEVER SE-150, Nissan Chemicals) followed by rubbing in order to promote unidirectional molecular alignment of liquid crystals. High quality of unidirectional alignment can be seen in polarized light microscopy images of the cell (Figure S11).

Measurements

The polarized optical microscopy investigations were performed using a microscope BX51 (Olympus). The absorption and transmittance spectra were measured using a spectrometer HR2000+ (Ocean Optics). For the polarized light absorbance measurements, spectrometer was equipped with a rotatable polarizer (Glan-Taylor prism). Temperature of the liquid crystal cells was controlled by using heating stage HS82 (Mettler Toledo), while the temperature controller equipped with magnetic stirrer (Quantum Northwest qpod2e) was used for solution studies.

As light source for operation of molecular motors, a mercury lamp (Spectroline) with a 312 nm filter (I~2.5mW/cm^2) was used. The intensity of the light was measured using a power meter PM-100D (Thorlabs).

Kinetic study

The thermal helix inversion kinetics at different temperatures was measured using UV-Vis spectroscopy. The quartz cells filled with mixture ZLI1695/Motor 1 – 4 were irradiated at 312 nm until the photostationary state was reached. Composition of photostationary (*cis*-unstable/*trans*-stable ratio) states of motors 1 – 4 were determined by NMR spectroscopy as 72/28, 87/13, 74/26, and 77/23, respectively. Then the cells were placed on a heating stage at a certain temperature for absorbance spectra recording. The thermal helix inversion rates (k) were determined by least-squares curve fittings (see Figures S2-S6) using rate equation (2) of a single-species irreversible reaction (1).

$$(cis - unstable) \xrightarrow{k} (cis - stable) \quad (1)$$

$$[cis - unstable] = [cis - unstable]_o e^{-kt} \quad (2)$$

Using the Eyring equation (3) for the set of rate constants (k) measured at different temperatures we calculated ΔH^*, ΔS^*, and ΔG^* (4) by plotting ln(k/T) vs 1/T according to equation (5).

$$k = \frac{k_B T}{h} e^{-\Delta G^*/RT} \quad (3)$$

$$\Delta G^* = \Delta H^* - T\Delta S^* \quad (4)$$

$$ln\frac{k}{T} = ln\frac{k_B}{h} + \frac{\Delta S^*}{R} - \frac{\Delta H^*}{R}\left(\frac{1}{T}\right) \quad (5)$$

Calculations

The structures of motors 1-4 were optimized in *trans-, cis*-stable-, and *cis*-unstable conformations, using density functional theory with PBE0 functional[3] and 6-311G(d,p)++ basis set. A conductor-like polarizable continuum model (C-PCM)[4] was used to model heptane solvent with ε = 1.92 and solvent molecule probe radii R = 3.125Å. In addition, transition state structures corresponding to the two helix inversion steps (*cis*-unstable → *cis*-stable and *trans*-unstable → *trans*-stable) were identified in all cases. Normal mode analysis was performed to prove character of all stationary points as well as evaluate thermochemistry at 298.15K

temperature. Optimized structures of all calculated conformations for motors 1, 2, 3 and 4 are uploaded as a separate archive file to the SI.

Vertical excitation spectra calculations were performed at the optimized geometries with time-dependent density functional theory (TD-DFT) method, PBE0 functional and 6-311G(d,p)++ basis set. The same C-PCM solvent model of heptane with $\varepsilon_\infty = 1.92$ was also used in spectra calculations.[5] Vertical excitation energies, orientations of molecular dipole moments, $S^0 \rightarrow S^1$ transition dipole moments (TDMs) as well as minimum moment of inertia (MOIs) were extracted from those calculations. The GAMESS(US) quantum chemistry package[6] was used to perform optimizations and spectra calculations.

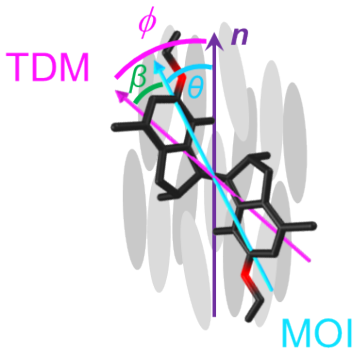

Scheme S2. Schematic representation of the molecular motor 1 (*trans* stable state) embedded into liquid crystals (grey ellipses) with unidirectional molecular orientation given by director **n**. TDM and MOI represent transition dipole moment and minimum moment of inertia, respectively.

The following equation was used to analyze an alignment direction and alignment quality of molecular motors embedded into unidirectionally oriented LC medium:[7,8]

$$S_\varphi = (A_\parallel - A_\perp)/(A_\parallel + 2A_\perp) = S_\theta S_\beta = <0.5(3\cos^2\theta - 1)>(0.5(3\cos^2\beta - 1)),$$

where where $A_\parallel$ and $A_\perp$— the absorbance of light polarized parallel and perpendicular to the liquid crystal alignment direction (**n**), S_φ is dichroic order parameter estimated from polarized UV-Vis measurements, φ is angle between LC director **n** and TDM; S_θ is an molecular order parameter defined by θ angle between MOI and **n**; S_β is TDM order parameter and β angle between TDM and MOI of the motor (extracted from TD-DFT calculations). From the equation,

S equals to 1 when TDMs are in perfect parallel alignment with liquid crystal molecules, and –0.5 if for perpendicular alignment. If TDMs are randomly orientated, S_φ reaches 0. The equation also shows that the dichroic order parameter consists of two contributors which relate to the angles θ and β as depicted in Scheme S2.

Table S1. Order parameters of the Motors 1 – 4 in three different states introduced into unidirectionally aligned nematic host.

	trans				***cis*-unstable**				***cis*-stable**			
	**1**	**2**	**3**	**4**	**1**	**2**	**3**	**4**	**1**	**2**	**3**	**4**
S_ϕ	0.11	0.22	0.22	0.68	0.01	-0.16	-0.21	0.28	0.05	-0.09	-0.26	0.52
S_β	0.80	0.72	0.69	0.99	0.98	-0.33	-0.42	0.99	0.99	-0.49	-0.29	0.99
S_θ	0.14	0.32	0.32	0.68	0.01	0.48	0.51	0.28	0.05	0.18	0.90	0.53
ϕ (°)	50.2	45.8	46.0	27.7	54.4	61.5	64.2	43.8	52.6	58.4	66.4	34.3
β (°)	21.6	25.4	27.2	3.4	5.9	70.6	76.9	0.8	3.1	87.0	67.9	2.7
θ (°)	49.1	42.5	42.2	27.5	54.4	36.2	34.9	43.7	52.7	47.7	14.8	34.2

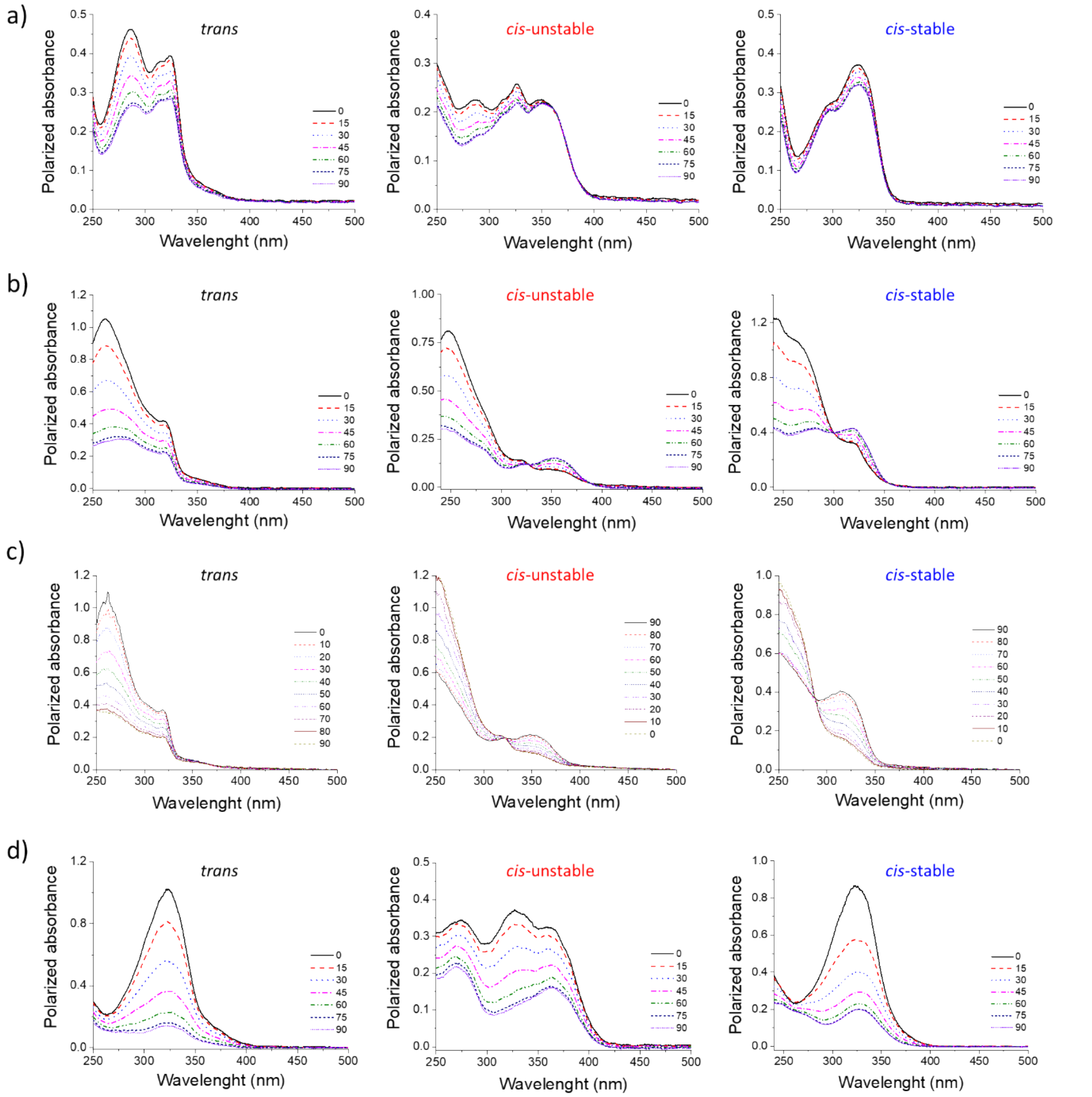

Figure S1. Polarized absorbance spectra of racemic mixtures of motor 1 (a), 2 (b), 3 (c), and 4 (d) embedded in unidirectional aligned liquid crystal host. The angle (from 0° to 90°) of light polarization with respect to molecular orientation of liquid crystals is shown for each spectrum.

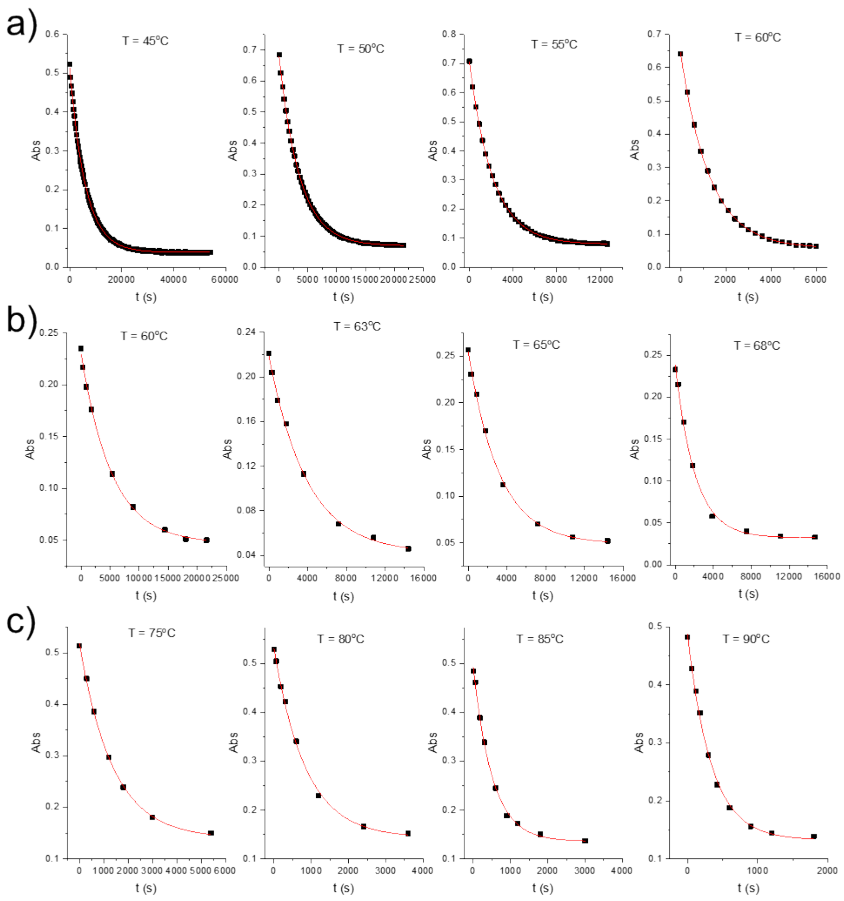

T (ºC)	k (s^{-1})	SE
45	1.68×10^{-4}	3.4×10^{-7}
50	2.81×10^{-4}	6.6×10^{-7}
55	4.68×10^{-4}	1.3×10^{-6}
60	7.80×10^{-4}	1.0×10^{-7}

T (ºC)	k (s^{-1})	SE
60	1.89×10^{-4}	9.6×10^{-6}
63	2.54×10^{-4}	1.5×10^{-5}
65	3.12×10^{-4}	1.8×10^{-5}
68	4.82×10^{-4}	2.8×10^{-5}

T (ºC)	k (s^{-1})	SE
75	7.31×10^{-4}	2.6×10^{-5}
80	1.17×10^{-3}	5.3×10^{-5}
85	1.95×10^{-3}	9.2×10^{-5}
90	2.95×10^{-3}	1.35×10^{-4}

Figure S2. Kinetic traces of thermal relaxation of cis-unstable state of motor 1 in heptane (a), nematic state of liquid crystals (b), and isotropic state of the liquid crystals (c). Rate constants are gathered in the tables on the right. SE corresponds to standard error.

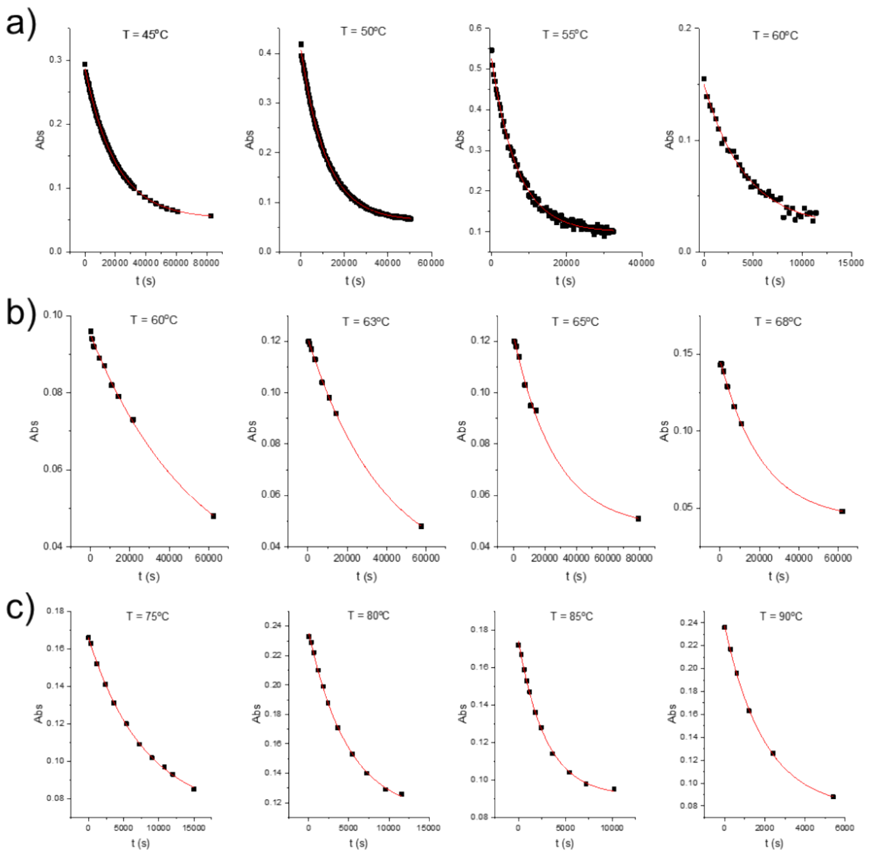

T (ºC)	k (s^{-1})	SE.
45	4.91×10^{-5}	2.8×10^{-7}
50	8.57×10^{-5}	2.7×10^{-7}
55	1.46×10^{-4}	2.3×10^{-6}
60	2.39×10^{-4}	1.4×10^{-5}

T (ºC)	k (s^{-1})	SE
60	1.88×10^{-5}	2.0×10^{-6}
63	2.61×10^{-5}	1.1×10^{-6}
65	3.78×10^{-5}	3.8×10^{-6}
68	4.69×10^{-5}	2.4×10^{-6}

T (ºC)	k (s^{-1})	SE
75	1.27×10^{-4}	4.9×10^{-6}
80	2.04×10^{-4}	9.1×10^{-6}
85	3.42×10^{-4}	1.3×10^{-5}
90	5.04×10^{-4}	2.4×10^{-5}

Figure S3. Kinetic traces of thermal relaxation of cis-unstable state of motor 2 in heptane (a), nematic state of liquid crystals (b), and isotropic state of the liquid crystals (c). Rate constants are gathered in the tables on the right. SE corresponds to standard error.

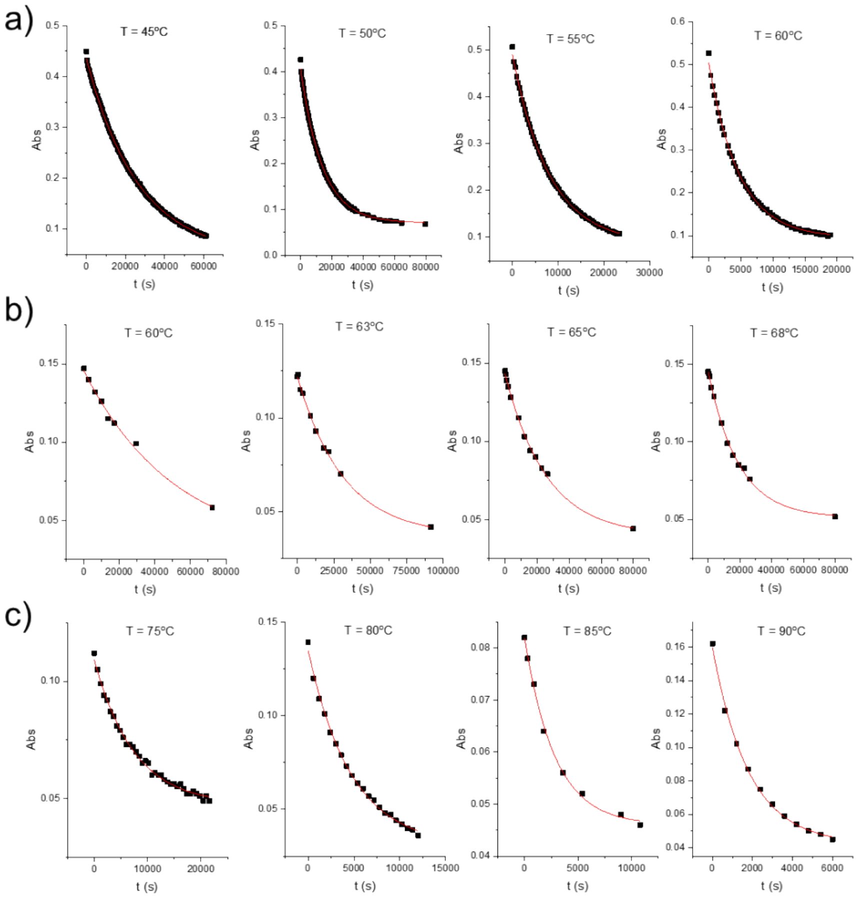

T (ºC)	k (s^{-1})	SE
45	3.82×10^{-5}	1.7×10^{-7}
50	7.22×10^{-5}	4.9×10^{-7}
55	1.25×10^{-4}	1.0×10^{-6}
60	2.13×10^{-4}	2.6×10^{-6}

T (ºC)	k (s^{-1})	SE
60	1.96×10^{-5}	3.3×10^{-6}
63	3.17×10^{-5}	1.7×10^{-6}
65	3.87×10^{-5}	1.6×10^{-6}
68	5.19×10^{-5}	2.3×10^{-6}

T (ºC)	k (s^{-1})	SE
75	1.34×10^{-4}	5.2×10^{-6}
80	2.13×10^{-4}	9.2×10^{-6}
85	3.53×10^{-4}	2.4×10^{-5}
90	5.50×10^{-4}	2.8×10^{-5}

Figure S4. Kinetic traces of thermal relaxation of cis-unstable state of motor 3 in heptane (a), nematic state of liquid crystals (b), and isotropic state of the liquid crystals (c). Rate constants are gathered in the tables on the right. SE corresponds to standard error.

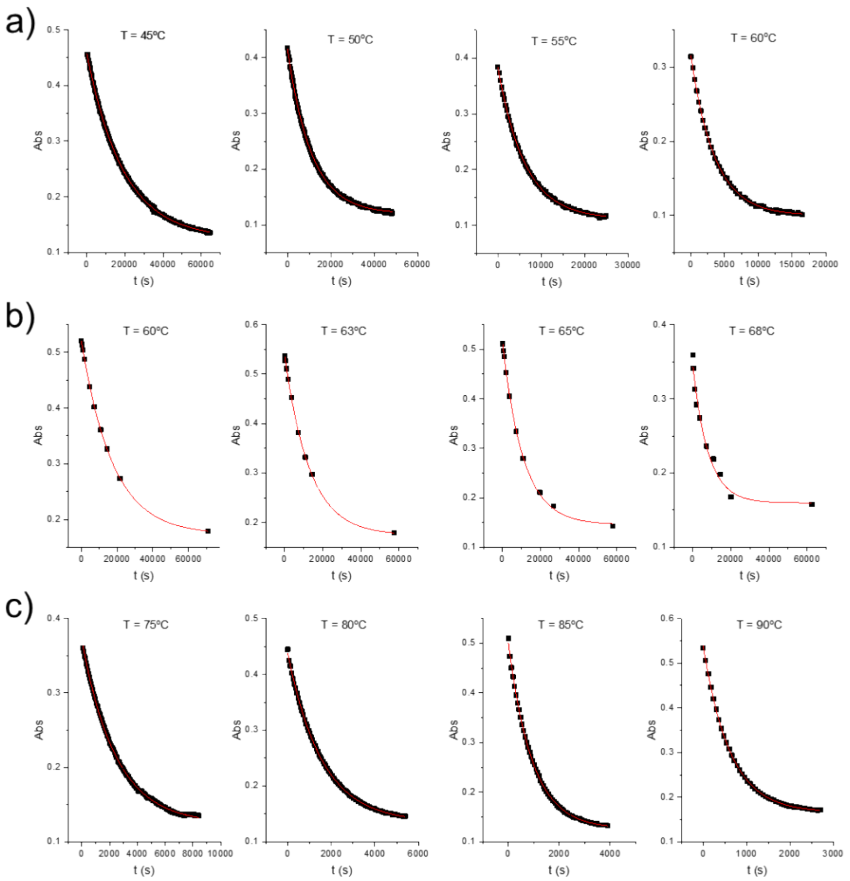

a)

T (ºC)	k (s^{-1})	SE
45	5.21×10^{-5}	1.6×10^{-7}
50	9.06×10^{-5}	3.5×10^{-7}
55	1.61×10^{-4}	8.1×10^{-7}
60	2.83×10^{-4}	1.7×10^{-6}

b)

T (ºC)	k (s^{-1})	SE
60	5.27×10^{-5}	4.6×10^{-7}
63	3.59×10^{-5}	1.1×10^{-6}
65	9.12×10^{-5}	2.6×10^{-6}
68	1.36×10^{-4}	3.4×10^{-5}

c)

T (ºC)	k (s^{-1})	SE
75	4.07×10^{-4}	2.3×10^{-6}
80	6.37×10^{-4}	2.6×10^{-6}
85	1.06×10^{-3}	6.2×10^{-5}
90	1.63×10^{-3}	7.3×10^{-5}

Figure S5. Kinetic traces of thermal relaxation of cis-unstable state of motor 4 in heptane (a), nematic state of liquid crystals (b), and isotropic state of the liquid crystals (c). Rate constants are gathered in the tables on the right. SE corresponds to standard error.

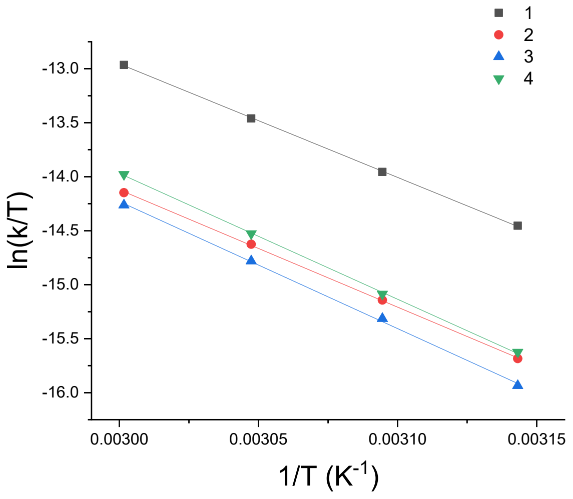

Figure S6. Eyring plot of THI of motors 1 – 4 in heptane.

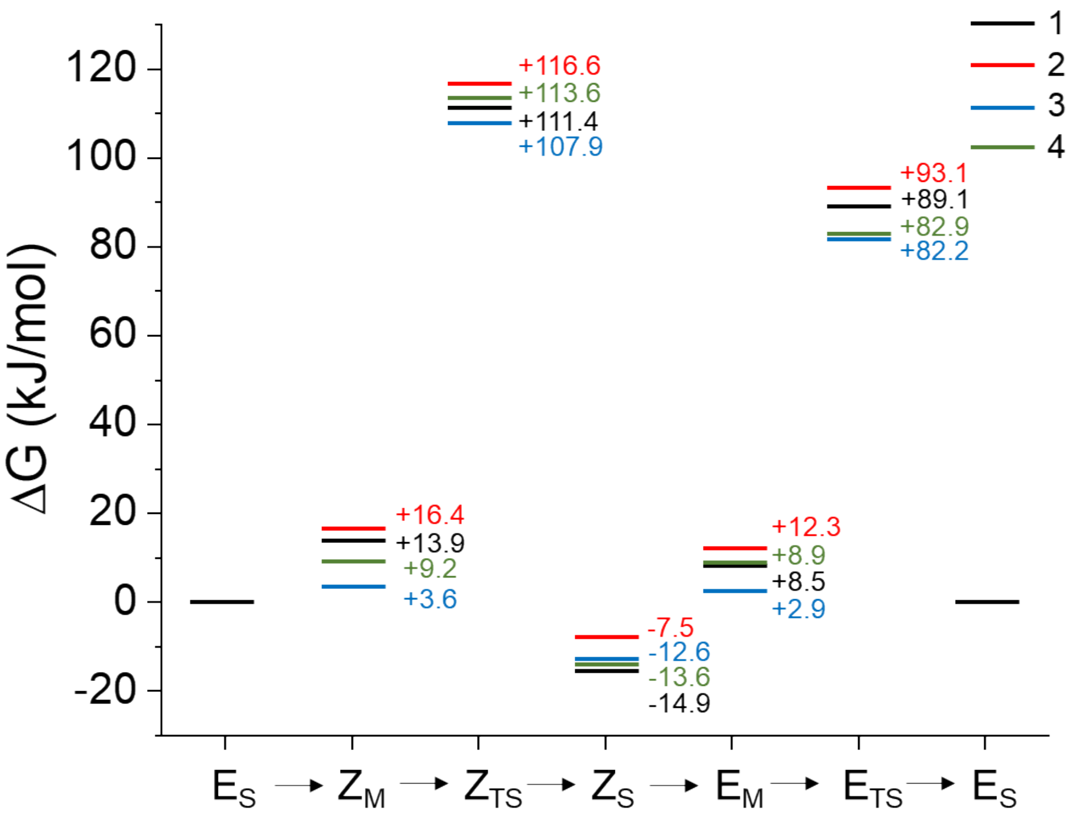

Figure S7. Calculated relative Gibbs free energies of motors 1 – 4. E_S – stable *trans*, Z_M – metastable *cis*, Z_{TS} - transition state *cis*, E_M – metastable *trans*, E_{TS} - transition state *trans*.

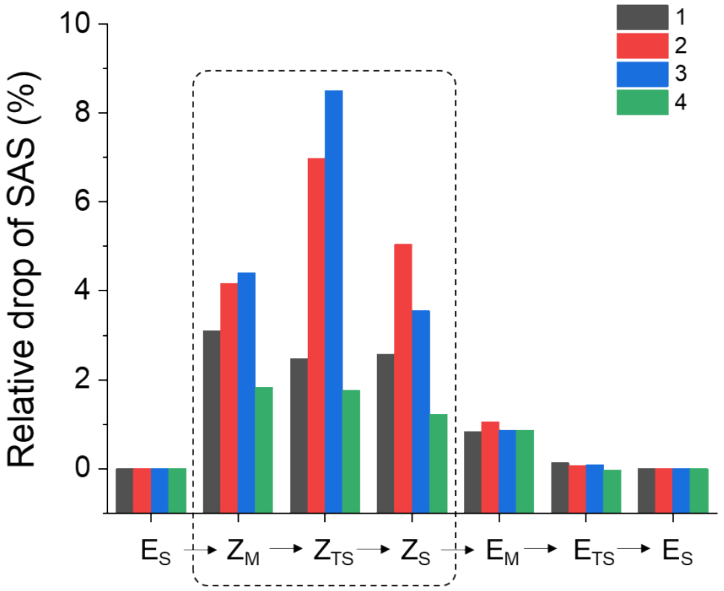

Figure S8. Relative reduction of solvent accessible surface area during rotation cycle of molecular motors 1 – 4. E_S – stable *trans*, Z_M – metastable *cis*, Z_{TS} - transition state *cis*, E_M – metastable *trans*, E_{TS} - transition state *trans*. The first thermal helix inversion step is indicated by dashed box.

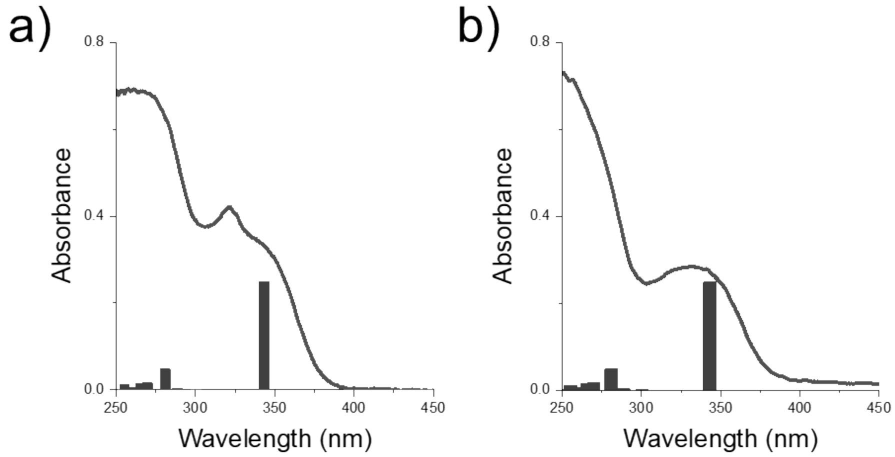

Figure S9. Absorbance spectra of *trans*-unstable rich state of motor 2 (a) and motor 3 (b) measured in liquid crystal host. Bars correspond to the most intense electronic transition calculated by using DFT.

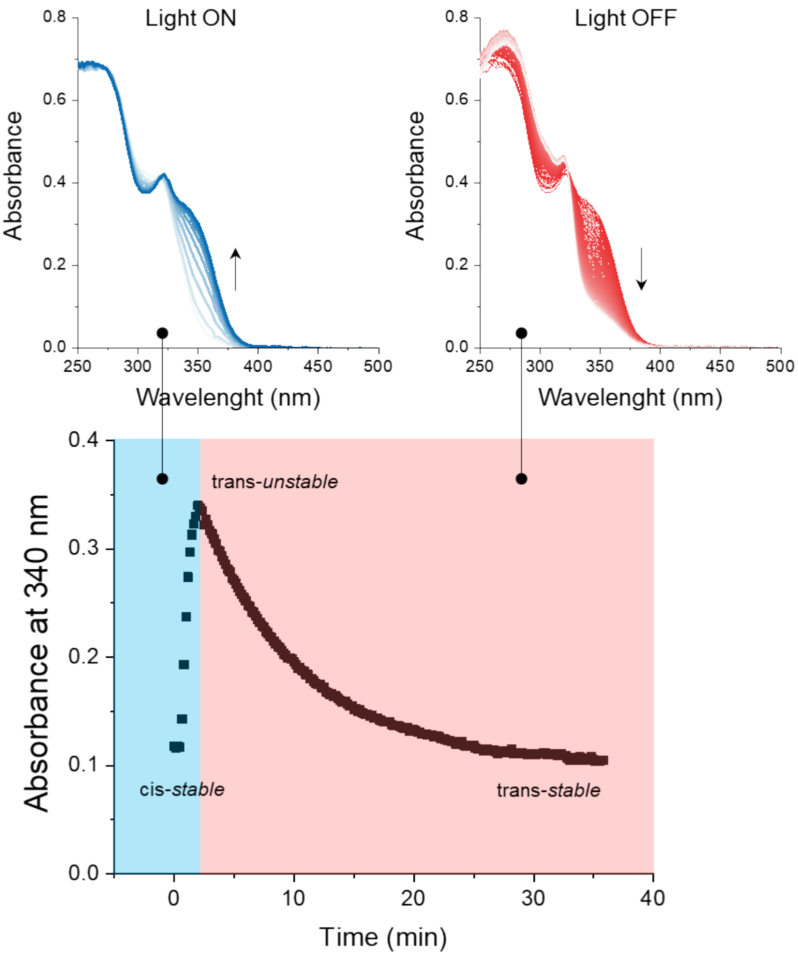

Figure S10. Absorbance spectra (top) of photo-induced (312 nm UV light) cis-*stable*- to trans-*unstable* state transition followed by its thermal relaxation to trans-*stable* state of motor 2 and corresponding kinetic profiles (bottom). Spectra measured in liquid crystal quartz cell at room temperature. The half-life of trans-unstable state was estimated as 5.8 minutes. Free activation energy 88.5 kJ/mol.

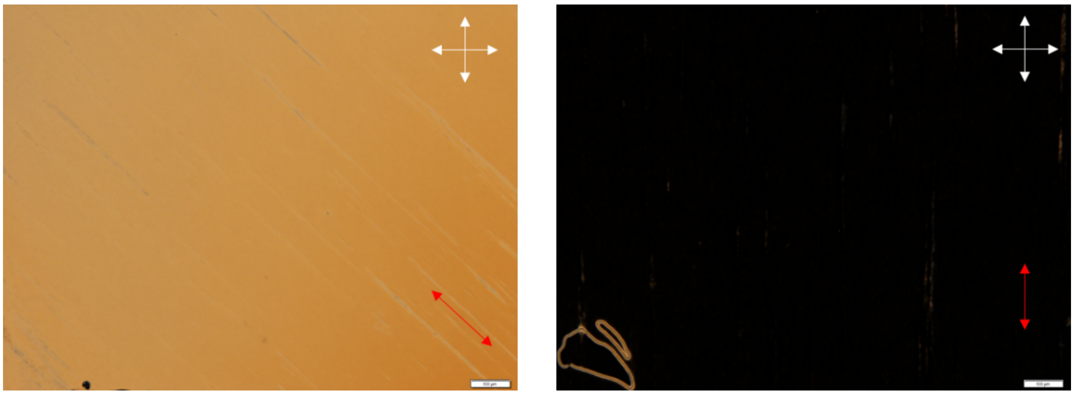

Figure S11. Polarized optical image of the liquid crystal cell filled with 1/ZLI1695 mixture. Images indicate unidirectional liquid crystals alignment. White arrows indicate the analyser and the polarizer. Red arrow indicates the rubbing direction, i.e. the direction of favoured molecular alignment. Scale bar is 100 µm.

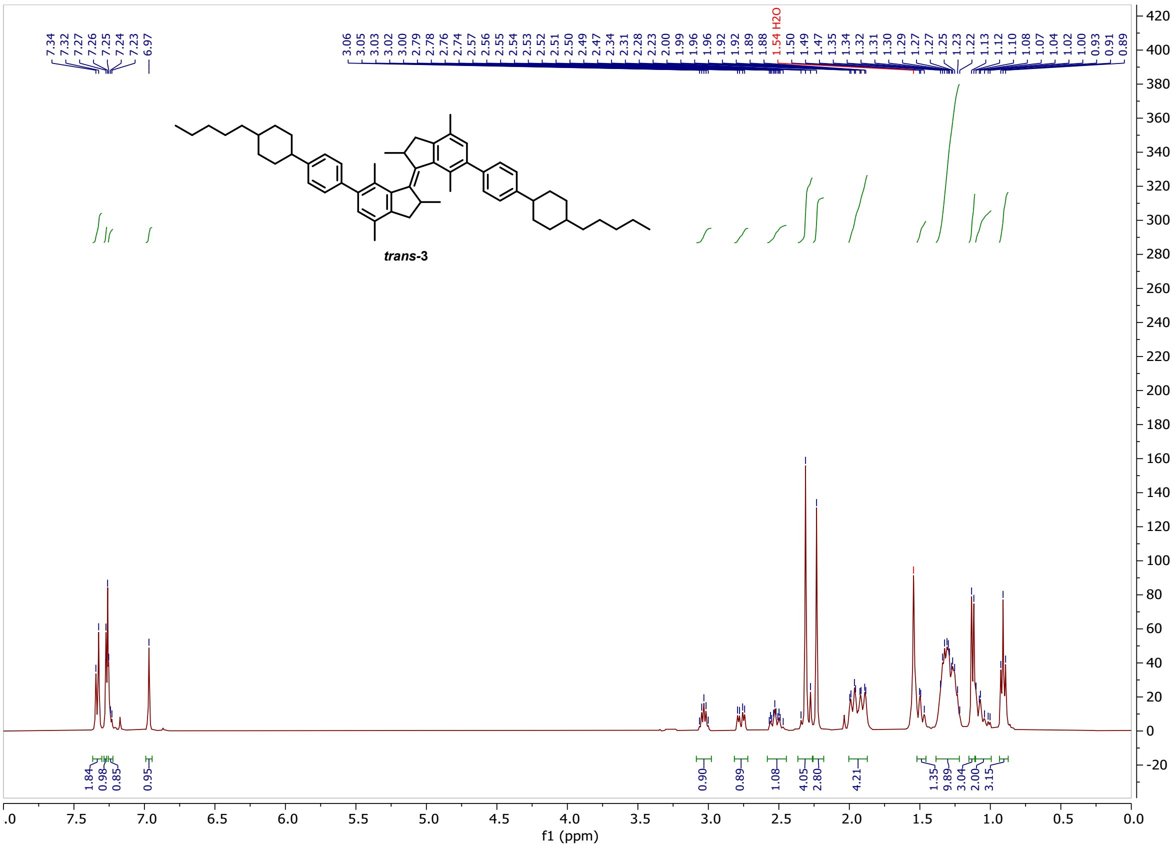

Figure S12. ^{1}HNMR spectrum (400 MHz, $CDCl_3$) of *trans*-3.

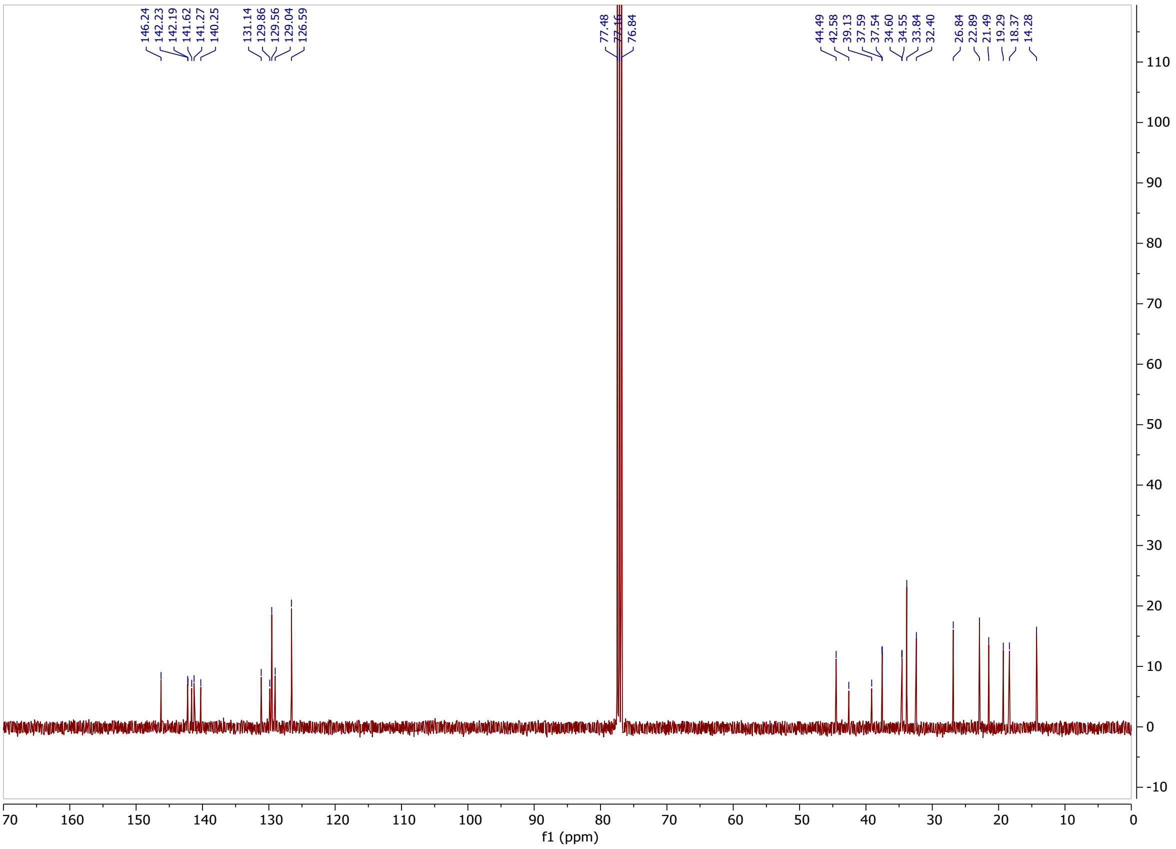

Figure S13. ^{13}C NMR spectrum (101 MHz, $CDCl_3$) of motor *trans*-3.

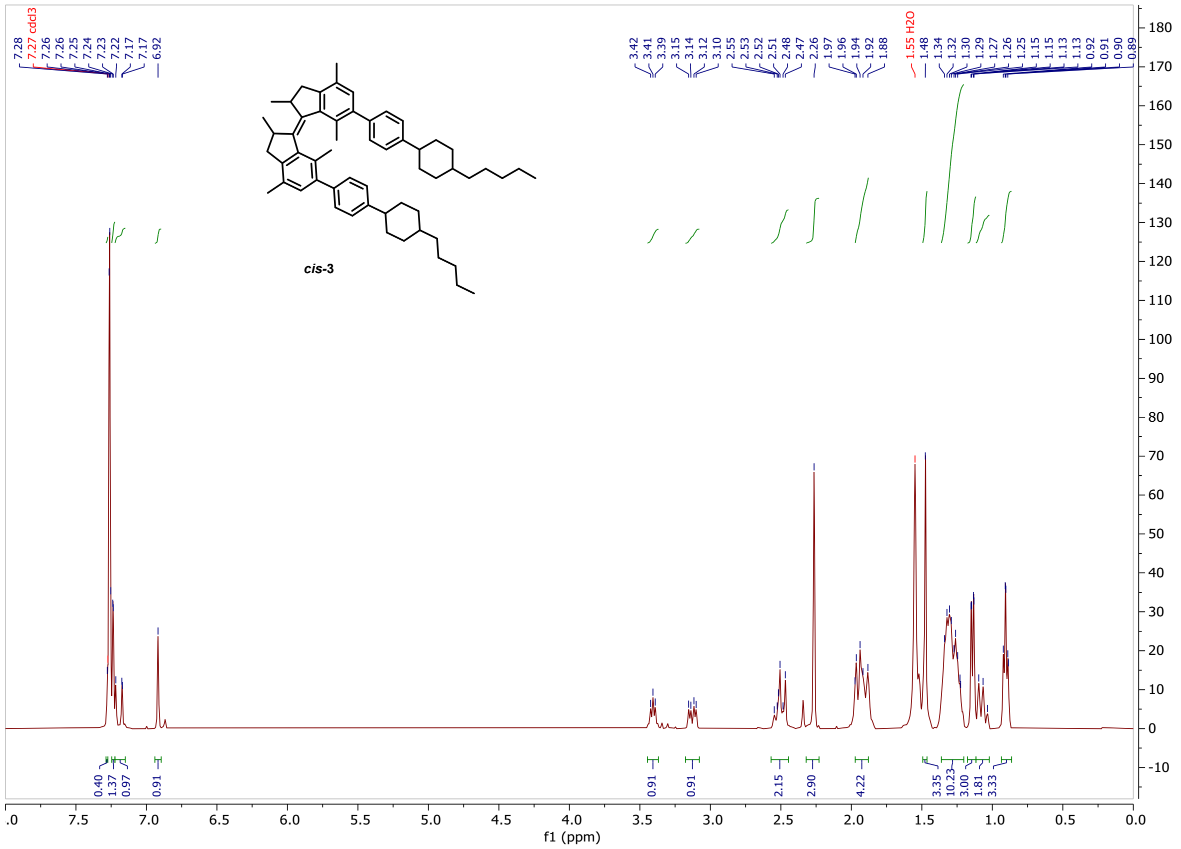

Figure S14. ^{1}HNMR spectrum (400 MHz, $CDCl_3$) of motor *cis*-3.

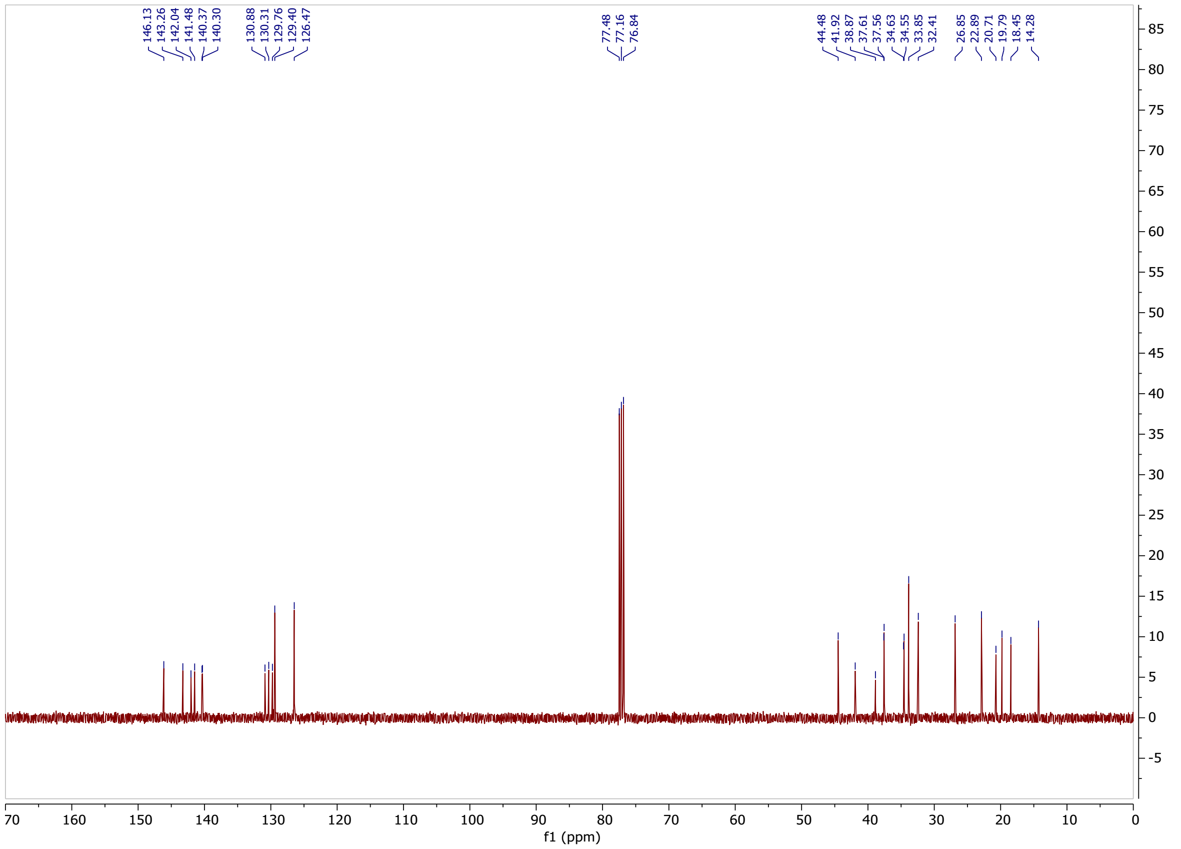

Figure S15. ^{13}C NMR spectrum (101 MHz, $CDCl_3$) of motor *cis*-3.

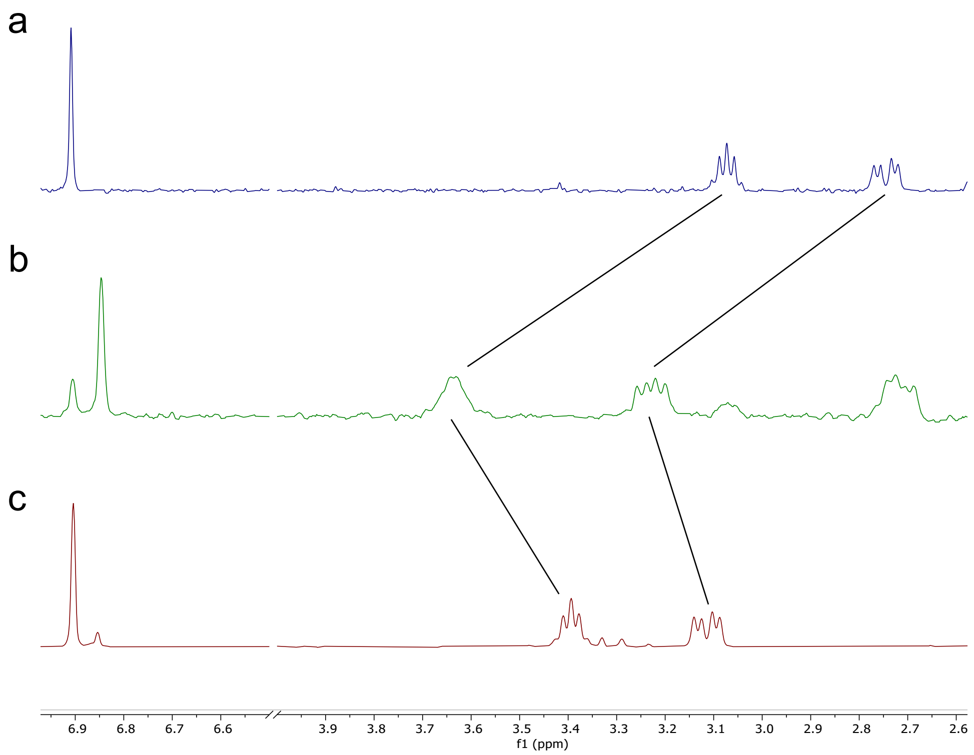

Figure S16. Partial ^{1}H-NMR of 3 (C_6D_{12}, 20 °C) (a) stable *trans*-3, before irradiation (λ=312nm); (b) after irradiation; and (c) stable *cis*-3.

References

1. Ryabchun, A.; Lancia, F.; Chen, J.; Morozov, D.; Feringa, B. L.; Katsonis, N., Helix Inversion Controlled by Molecular Motors in Multistate Liquid Crystals. *Adv. Mater.* **2020**, 32 (47), 2004420.

2. Neubauer, T. M.; van Leeuwen, T.; Zhao, D.; Lubbe, A. S.; Kistemaker, J. C. M.; Feringa, B. L., Asymmetric Synthesis of First Generation Molecular Motors. *Org. Lett.* **2014**, 16 (16), 4220-4223.

3. Adamo, C.; Barone, V. Toward Reliable Density Functional Methods without Adjustable Parameters: The PBE0 Model. *J. Chem. Phys.* **1999**, *110*, 6158–6170.

4. Cossi, M.; Rega, N.; Scalmani, G.; Barone, V. Energies, Structures, and Electronic Properties of Molecules in Solution with the C-PCM Solvation Model. *J. Comput. Chem.* **2003**, *24*, 669–681.

5. Cossi, M.; Barone, V. Time-Dependent Density Functional Theory for Molecules in Liquid Solutions. *J. Chem. Phys.* **2001**, *115*, 4708–4717.

6. Schmidt, M. W.; Baldridge, K. K.; Boatz, J. A.; Elbert, S. T.; Gordon, M. S.; Jensen, J. H.; Koseki, S.; Matsunaga, N.; Nguyen, K. A.; Su, S.; *et al.* General Atomic and Molecular Electronic Structure System. *J. Comput. Chem.* **1993**, *14*, 1347–1363.

7. Li, Y., Chen, Y., Li, H., Liu, C., Li, L., Quan, Y., Cheng, Y. Achiral Dichroic Dyes-mediated Circularly Polarized Emission Regulated by Orientational Order Parameter through Cholesteric Liquid Crystals. *Angew. Chem. Int. Ed.* **2023**, *62*, e202312159.

8. Sims, M. T. Dyes as guests in ordered systems: current understanding and future directions. *Liq. Cryst.*, **2016**, *43*, 2363-2374.
